# Enrichment of Mutations in Multiple DNA Sequences Using COLD-PCR in Emulsion

**DOI:** 10.1371/journal.pone.0051362

**Published:** 2012-12-06

**Authors:** Elena Castellanos-Rizaldos, Coren Audrey Milbury, G. Mike Makrigiorgos

**Affiliations:** 1 Division of DNA Repair and Genome Stability, Department of Radiation Oncology, Dana-Farber Cancer Institute, Harvard Medical School, Boston, Massachusetts, United States of America; 2 Division of Medical Physics and Biophysics, Department of Radiation Oncology, Dana-Farber Cancer Institute and Brigham and Women's Hospital, Harvard Medical School, Boston, Massachusetts, United States of America; Saint Louis University, United States of America

## Abstract

**Background:**

Multiplex detection of low-level mutant alleles in the presence of wild-type DNA would be useful for several fields of medicine including cancer, pre-natal diagnosis and infectious diseases. COLD-PCR is a recently developed method that enriches low-level mutations during PCR cycling, thus enhancing downstream detection without the need for special reagents or equipment. The approach relies on the differential denaturation of DNA strands which contain Tm-lowering mutations or mismatches, versus ‘homo-duplex’ wild-type DNA. Enabling multiplex-COLD-PCR that can enrich mutations in several amplicons simultaneously is desirable but technically difficult to accomplish. Here we describe the proof of principle of an emulsion-PCR based approach that demonstrates the feasibility of multiplexed-COLD-PCR within a single tube, using commercially available mutated cell lines. This method works best with short amplicons; therefore, it could potentially be used on highly fragmented samples obtained from biological material or FFPE specimens.

**Methods:**

Following a multiplex pre-amplification of *TP53* exons from genomic DNA, emulsions which incorporate the multiplex product, PCR reagents and primers specific for a given *TP53* exon are prepared. Emulsions with different *TP53* targets are then combined in a single tube and a fast-COLD-PCR program that gradually ramps up the denaturation temperature over several PCR cycles is applied (temperature-tolerant, TT-fast-eCOLD-PCR). The range of denaturation temperatures applied encompasses the critical denaturation temperature (T_c_) corresponding to all the amplicons included in the reaction, resulting to a gradual enrichment of mutations within all amplicons encompassed by emulsion.

**Results:**

Validation for TT-fast-eCOLD-PCR is provided for *TP53* exons 6–9. Using dilutions of mutated cell-line into wild-type DNA, we demonstrate simultaneous mutation enrichment between 7 to 15-fold in all amplicons examined.

**Conclusions:**

TT-fast-eCOLD-PCR expands the versatility of COLD-PCR and enables high-throughput enrichment of low-level mutant alleles over multiple sequences in a single tube.

## Introduction

Detection of low-level mutant alleles in the presence of wild-type DNA is important in several fields of medicine, including cancer, pre-natal diagnosis and infectious diseases [1]. A number of approaches have been described that enrich the abundance of low-level mutant allelic burden present in biological specimens, so that mutations can subsequently be detected [1]. These include electrophoretic or enzymatic approaches, which require multiple reagents or specialized equipment. Co-amplification of major and minor DNA alleles at Lower Denaturation temperature (COLD-PCR) represents a PCR-based technique that enriches mutation-containing sequences by adjusting the denaturation temperature to within ∼0.3°C of a critical temperature (T_c_). This enables preferential amplification of mutated sequences during the PCR cycling program irrespective where the mutation lies on the amplicon [2]. A main advantage of this procedure is its simplicity, since it does not require new instrumentation, special reagents or additional processing. This makes COLD-PCR accessible to different laboratory settings [3] that employ a variety of downstream mutation detection assays including high resolution melting, HRM [4]–[8], Sanger sequencing [9]–[12], mass-spectrometry [2], real time PCR [13], [14] and RFLP analysis [15].

**Figure 1:**
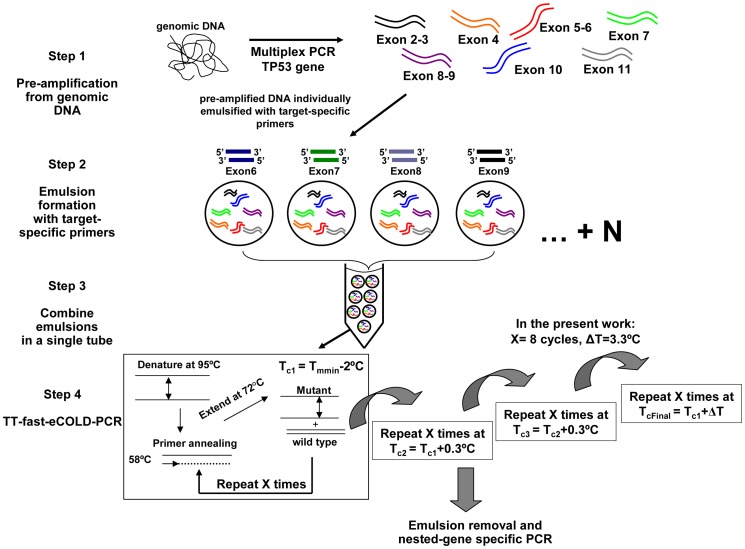
Temperature-tolerant-fast-COLD-PCR in emulsion: Overview of the steps involved. Multiplex pre-amplification from genomic DNA; emulsification with gene-specific primers; mixing into a single tube; and temperature-tolerant emulsion-based fast-COLD-PCR.

A COLD-PCR drawback is that it is not easily scalable for enriching mutations in diverse amplicons simultaneously, since generally, a different T_c_ applies to each amplicon. The requirement for determining T_c_ with a precision of ∼0.3°C means that T_c_ is subject to variations due to different brand thermo-cyclers, well-to-well temperature fluctuations or reagent conditions [16]. For this reason, we recently developed temperature-tolerant-COLD-PCR (TT-COLD-PCR), a modified form of COLD-PCR that applies to all COLD-PCR formats described, [2], [17]. TT-COLD-PCR relaxes the stringency on T_c_ and allows sequences of different T_c_ to be amplified using a single thermo-cycling program [16]. In TT-COLD-PCR, amplification starts at a lower T_c_ and increases gradually over several PCR cycles, spanning an overall 2–3°C temperature window. This enables enrichment of mutations in all amplicons whose T_c_ lies within the temperature window [16]. Yet individual DNA targets still need to be amplified in separate tubes using TT-COLD-PCR and adaptation to multiplex formats is difficult in view of primer-primer interactions. Multiple primer interactions can be even more problematic for COLD-PCR compared to conventional PCR, in view of the low denaturation temperatures applied that may enhance primer-dimer formation and non-specific amplification products.

In this work we demonstrate that the problem of multiplexing COLD-PCR can be overcome by employing temperature-tolerant amplification in emulsion. We describe a COLD-PCR protocol that allows simultaneous enrichment of mutations in multiple DNA targets of different Tm in a single tube by applying a temperature-tolerant cycling protocol in emulsion. PCR amplification inside water-in-oil emulsion enables compartmentalization that eliminates multiple primer interactions when co-amplifying complex DNA templates [18]–[20]. We demonstrate the application of temperature-tolerant fast-COLD-PCR in emulsion (TT-fast-eCOLD-PCR, **Figure 1**) by enriching mutations in four exons of the *TP53* gene, in a single tube.

**Table 1:** Cell lines used in the present study.

Gene and amplicon		Cell line	Mutation (nt)	Mutation (aa)
*TP53*	Exon 6	SNU-182	c.644G>T	p.S215I
	Exon 7	HCC2157	c.742C>T	p.R248W
	Exon 8	SW480	c.818G>A	p.R273H
	Exon 9	SW480	c.925C>T	p.P309S

**Table 2:** Primer sequences used in this study.

Gene and amplicons	PCR Round	Primer sequences (5' to 3')^1^		Amplicon size (bp)
***TP53*** ** Exon 2–11** 2	Pre-amplification (multiplex reaction)	Exon 2–3	ATGCTGGATCCCCACTTTTC (*F*)	350
			GACCAGGTCCTCAGCCC (*R*)	
		Exon 4	GACAAGGGTTGGGCTGG (*F*)	486
			CCAAAGGGTGAAGAGGAATC (*R*)	
		Exon 5–6	TCTTTGCTGCCGTCTTCC (*F*)	517
			AGGGCCACTGACAACCAC (*R*)	
		Exon 7	TGCTTGCCACAGGTCTCC (*F*)	235
			GTCAGAGGCAAGCAGAGGC (*R*)	
		Exon 8–9	GGACAGGTAGGACCTGATTTCC (*F*)	441
			AAACAGTCAAGAAGAAAACGGC (*R*)	
		Exon 10	AACTTGAACCATCTTTTAACTCAGG (*F*)	243
			GGAATCCTATGGCTTTCCAAC (*R*)	
		Exon 11	AGGGGCACAGACCCTCTC (*F*)	222
			AGACCCAAAACCCAAAATGG (*R*)	
***TP53*** ** Exon 6–9**	TT-*fast-*COLD-ePCR	Exon 6^3^	TCACTGATTGCTCTTAGGTCT *(F)*	144
			GTTGCAAACCAGACCTC *(R)*	
		Exon 7	TCTTGGGCCTGTGTTAT *(F)*	122
			AGTGTGATGATGGTGAG *(R)*	
		Exon 8^3,4^	TTGCTTCTCTTTTCCTAT *(F)*	115
			GCGGAGATTCTCTTC *(R)*	
		Exon 9^3^	AGGGTGCAGTTATGCCTCAG *(F)*	114
			TCTCCATCCAGTGGTTTCTTC *(R)*	

1 Oligonucleotides (F) forward or (R) reverse.

2 Oligonucleotide sequences described before [21], [22].

3 Oligonucleotides from Castellanos et al. [16].

4 Primer sequences described previously [2], [17].

## Materials and Methods

Reference human genomic DNA obtained from Promega, Inc. (catalog number G1471, Madison, WI, USA) was used as wild type control and for preparing dilutions with mutant cell lines. Genomic DNA from cell lines with *TP53* mutations in exons 6–9 was purchased from ATCC (Manassas, VA, USA), **Table 1**. A genomic DNA mixture containing 5% mutant DNA abundance, resulting from a combination of DNA from mutated cell lines into human male genomic DNA, was tested. To obtain a simplified genomic template for subsequent TT-fast-COLD-ePCR reactions, exons 2–11 of *TP53* gene were pre-amplified in a single multiplex-PCR reaction from genomic DNA as reported by Fredriksson [21] with minor modifications [22]. Briefly, multiplex reactions were performed in 15 μl final reaction volumes containing 1× Phusion™ high-fidelity (HF) buffer, 0.2 mmol/L (each) dNTPs, 0.1 μmol/L (each) primers, and 0.6 U Phusion™ high fidelity DNA polymerase (New England Biolabs Inc., Ipswich, MA), reported to have an error rate of 4.2×10^−7^. Primers were synthesized by Integrated DNA Technologies (IDT Inc., Coralville, IA, USA) and are summarized in **Table 2**.

**Table 3:** PCR thermocycling conditions utilized in the present work.

PCR type	Step	Conditions	*T*_a_°C
**Multiplex-PCR** 1 **(pre-amplification)**	Initial denaturation	98°C for 30 s	
	Thermocycling: 35 cycles	98°C for 10 s	Exon 2–11 *TP*53, 55°C
		55°C for 20 s	
		72°C for 10 s	
	Extension	72°C for 15 s	
	Stage 1 cycling: 5 times	95°C for 10 s	
		*T_a_* for 20 s	
		72°C for 10 s	
	Stage 2 cycling: 8 times	85.2°C for 10 s	
		*T_a_* for 20 s	
		72°C for 10 s	
	Stage 3 cycling: 8 times	85.5°C for 10 s	
		*T_a_* for 20 s	
		72°C for 10 s	
	Stage 4 cycling: 8 times	85.8°C for 10 s	
		*T_a_* for 20 s	
		72°C for 10 s	
	Stage 5 cycling: 8 times	86.1°C for 10 s	
		*T_a_* for 20 s	
		72°C for 10 s	
**TT-** ***fast*** **-COLD- PCR**	Stage 6 cycling: 8 times	86.4°C for 10 s	Exon 6–9 *TP*53, 58°C
		*T_a_* for 20 s	
		72°C for 10 s	
	Stage 7 cycling: 8 times	86.7°C for 10 s	
		*T_a_* for 20 s	
		72°C for 10 s	
	Stage 8 cycling: 8 times	87°C for 10 s	
		*T_a_* for 20 s	
		72°C for 10 s	
	Stage 9 cycling: 8 times	87.3°C for 10 s	
		*T_a_* for 20 s	
		72°C for 10 s	
	Stage 10 cycling: 8 times	87.6°C for 10 s	
		*T_a_* for 20 s	
		72°C for 10 s	
	Stage 11 cycling: 8 times	87.9°C for 10 s	
		*T_a_* for 20 s	
		72°C for 10 s	
	Stage 12 cycling: 8 times	88.2°C for 10 s	
		*T_a_* for 20 s	
		72°C for 10 s	
	Stage 13 cycling: 8 times	88.5°C for 10 s	
		*T_a_* for 20 s	
		72°C for 10 s	

1 Conditions from a previous study [22].

For emulsion-PCR/COLD-PCR, the aqueous phase of the reaction (50 µl volume) was prepared individually for each target amplicon with 0.2 µmol/L amplicon-specific primers (F and R), 1× Phusion™ Buffer, 0.2 mmol/L (each) dNTPs, 2 U of Phusion™ DNA polymerase, 1× LCGreen (Idaho Technology Inc., Salt Lake City, Utah, USA) and 2 µl of DNA template up to 50 µl with water. The oil phase of the reaction was prepared as described [23]–[25]; 4.5% Span 80 (product no. S6760, Sigma Aldrich, USA), 0.4% Tween 80 (product no. P1754, Sigma Aldrich, USA), 0.05% Triton X-100 (part no. T1001, Affymetrix, USA) were mixed with 100% mineral oil (product no. M5904, Sigma Aldrich) to 50 ml. Then, 300 µl of the oil phase was mixed with 50 µl of the aqueous phase in separate tubes for each of the target amplicons and vortexed for 5 min at high speed. 100 µl aliquots were used during the amplification reaction to ensure proper heat distribution.

**Figure 2:**
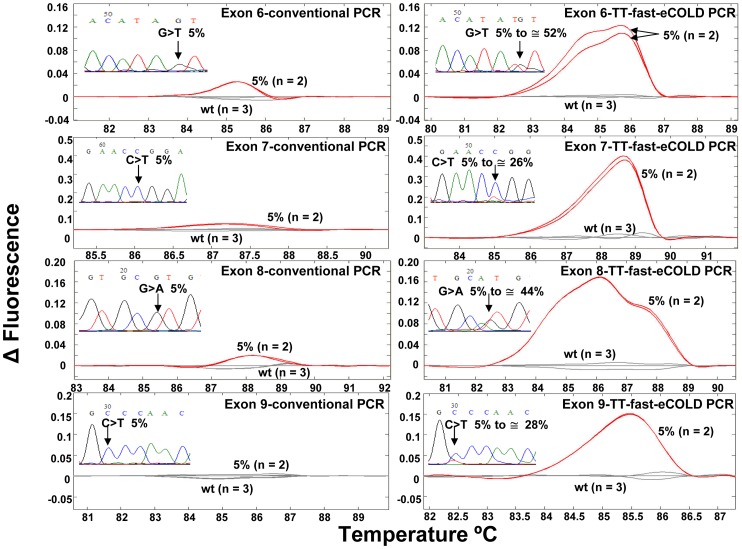
Temperature-tolerant COLD-PCR in emulsion, TT-fast-eCOLD-PCR: Enrichment of mutations in multiple DNA sequences in a single tube. A 5% mutation abundance was evaluated for *TP53* gene exons 6–9 by conventional PCR (left panels) and TT-fast-eCOLD-PCR (right panels). Duplicate experiments are depicted in each case. The enrichment of the mutations in all four exons is estimated from the chromatograms.

In preliminary experiments, the optimal amount of target DNA molecules per micelle was determined for each target amplicon and appropriate dilutions from the pre-amplification product were used as template for TT-fast-eCOLD-PCR. TT-fast-eCOLD-PCR was performed using the guidelines described, and with minor modifications, per **Table 3**. To retrieve amplified DNA following TT-fast-eCOLD-PCR, corresponding samples were transferred to 1.5 ml tubes and centrifuged for 5 min at 13,000 g [23], [24] to remove the upper oil phase. Then, samples were mixed with 1 ml of butanol **(**catalog. no. B7906, Sigma Aldrich, USA) by vortexing and further purified using the Qiagen purification kit according to manufacturer’s instructions [25]. To analyze the amplified DNA, following emulsion removal, recovered DNA was further amplified by conventional PCR using nested primers specific for each amplicon in the presence of 1X LCGreen intercalating dye. The products were analyzed using high resolution melting (HRM) with a Light Scanner HR96 system (Idaho Technologies, Inc.) and Sanger-sequenced at Eton Bioscience, Inc. (Cambridge, MA, USA). Sanger chromatograms were analyzed with BioEdit v7.1.3 (Ibis Biosciences, Abbott Laboratories, USA) to determine the approximate mutational load from the peak height as previously described [17]. All experiments were repeated multiple independent times.

To test the amplification efficiency during the emulsion PCR step, a control experiment was performed by performing a subsequent, nested real time PCR using LC-Green dye from the emulsion-PCR and comparing the real-time PCR threshold difference with and without the emulsion-PCR step. Furthermore, to ensure that amplification takes place solely within the emulsion and not in the aqueous phase, exonuclease I (*Exo*I, New England Biolabs, MA) was added to the reactions to eliminate the extracellular portion of the PCR primers. Experiments to validate the efficient degradation of PCR primers in the aqueous phase of the sample by *Exo*I were performed, with and without forming the emulsion (5 min vigorous vortexing versus gently mixing the oil and aqueous phase, respectively).

## Results

### Emulsion-PCR Validation

The present procedures yield formation of emulsions with diameters of 3–10 µm (**Figure S1**). We verified efficient amplification in emulsion (ePCR) by monitoring the product yield (Ct value) in a subsequent, nested real-time PCR reaction that utilizes diluted ePCR product as a template. In **Figure S2**, panel A shows 10 cycles PCR threshold difference in the nested PCR between emulsified-amplified DNA and versus an identically treated sample where the ePCR step was omitted. In panel B, we observe that, when *Exo*I is added, in the absence of emulsion there is no PCR product formed, presumably due to the anticipated ssDNA (primer) hydrolysis by *Exo*I. In contrast, when the emulsion is formed in the same samples by 5 min vigorous vortexing, subsequent addition of *Exo*I does not inhibit PCR, consistent with the expectation that the hydrophilic *Exo*I does not penetrate the emulsion. These data are consistent with amplification taking place within the emulsion efficiently and not in the extracellular space when *Exo*I is present. *Exo*I was added to the reactions described below to exclude amplification taking place outside emulsions.

### TT-fast-eCOLD-PCR for Individual Amplicons

**Figure S3** demonstrates that mutation enrichments of ∼7–15 fold are obtained for exons 6–9 when these are amplified as separate, single-amplicon reactions. This figure depicts TT-fast-eCOLD-PCR applied in emulsion, using as starting material genomic DNA containing 5% mutant DNA, and followed by Sanger sequencing of the final product. In contrast, when conventional PCR, instead of COLD-PCR, is applied to the same emulsions, the 5% mutant peaks are not visible in any of the chromatograms, indicating the efficient enrichment of mutations using the TT-fast-eCOLD-PCR cycling program.

### TT-fast-eCOLD-PCR for *TP53* Exons 6–9 in a Single Tube

To perform TT-fast-eCOLD-PCR in a single tube for all targets (**Figure 1**), *TP53* exons 6–9 were emulsified individually then combined into a single tube and cycled on an Eppendorf Mastercycler (Eppendorf Inc., Haupage, NY). The TT-fast-eCOLD-PCR program (**Table 3** **)** was designed to simultaneously amplify exons 6–9 of *TP53* gene as described earlier [16], and by spanning a range of T_c_ from 85.2°C–88.5°C, with 0.3°C temperature increments every eight cycles (initial T_c_ = lowest melting temperature of the amplicons tested minus 2°C). Fluorescent difference curve plots relative to wild type DNA become clearly detectable via HRM analysis in those amplicons generated by TT-fast-eCOLD-PCR, **Figure 2**. In contrast, HRM analysis of the same amplicons amplified by conventional PCR in emulsion does not show a signal distinct from the wild type profile. HRM results were further validated via Sanger sequencing, **Figure 2**. The data indicate that the mutant abundance displayed a ∼5–10 fold enrichment in all exons simultaneously when amplified by TT-fast-eCOLD-PCR. These results are comparable with the mutation enrichment obtained following one-round fast –COLD-PCR of single amplicons in solution [2]. Therefore, the use of the emulsion with the multiplex format of TT-fast-COLD-PCR does not significantly affect the mutation enrichment obtained.

It is noteworthy that when the same TT-fast-COLD-PCR cycling of the 4 exons is performed in solution, (i.e. in the absence of emulsion reagents or *Exo*I), Sanger sequencing depicts mutation enrichment in 3 out of 4 amplicons (exon 6, 8 and 9), while for exon 7 no amplification is obtained (**Figure S4**). The data indicate that multiple primers in solution lead to primer-primer interactions that inhibit efficient generation of the amplicon from exon 7. The compartmentalization provided by the emulsion reduces multiple primer interactions and overcomes this problem.

## Discussion

Since its original description, the development of PCR in emulsion has been exploited for several applications, such as quantification of DNA methylation [26], studying *in vitro* directed evolution [18], [19], [27], [28] or detection of rare variants at specific mutation ‘hotspot’ positions [29]. The data presented here provide proof of principle for one yet application of emulsion-PCR. By compartmentalizing COLD-PCR reactions and ramping-up the temperature gradually, multiple targets with different melting temperature (T_m_) and T_c_ are amplified and enriched simultaneously while preventing excessive primer and template interactions.

In principle, a high number of amplicons with T_c_ within the range covered by the amplification protocol can be co-amplified using COLD-PCR in emulsion. In practice the rate-limiting step is the initial pre-amplification stage plus the preparation of emulsions containing multiplex-PCR targets and discrete primers for each target. It is envisioned that micro-fluidics can overcome these limitations. Thus, the number of sequences can be scaled-up by employing recent developments that merge complex DNA templates with sequence-specific primers over millions of emulsions per experiment [19], [30]. These new engineering tools can facilitate the high-throughput adaptation of the TT-fast-eCOLD-PCR principle presented in this work. The mutation-enriched DNA can optionally be combined with high-throughput sequencing platforms to improve the reliability and lowest limit of mutation calling [31]. Alternatively, high throughput genotyping methods (MALDI) can be used as the endpoint detection system [32].

In the present manuscript we have shown the adaptation of fast-COLD-PCR format in emulsion. This form of COLD-PCR is only applicable to mutations that decrease the melting temperature of the amplicon (G:C>A:T, G:C>T:A, or Tm-reducing indels). Tm-reducing base substitutions account for the majority of mutations in cancer [33]. The adaptation of other forms of COLD-PCR (full- or ice-COLD-PCR) in emulsion, analyzing clinical samples fresh or in formalin (FFPE), is also desirable and will be addressed in future work.In summary, we demonstrated that emulsion-PCR overcomes the limitations of unwanted multiple-primer interactions during temperature-tolerant COLD-PCR and enables single-tube amplification and mutation enrichment for multiple target DNA sequences. This development facilitates multiplexed enrichment and detection of panels of clinically-relevant mutations to aid prognosis, diagnosis and management in heterogeneous cancer samples.

## Supporting Information

Figure S1 **Water in oil emulsion at 100× magnification with a Zeiss Axioimager Z1 fluorescent microscope (Carl Zeiss Microscopy, GmbH, Germany) after five minutes of vortexing.**
(TIF)

Figure S2 **Validation of emulsion-PCR.** **Panel A:** DNA amplified by emulsion PCR (ePCR) or where ePCR amplification was omitted, was evaluated by an additional nested, real-time qPCR using LCGreen (duplicate reactions). **Panel B:** Effect of adding exonuclease I (*Exo*I) in the sample prior to ePCR, with and without emulsion formation, as evaluated by nested, qPCR. ***Left*** **:** aqueous and oil phase without emulsion formation (gently mixed) with and without *Exo*I. ***Right*** **:** emulsion (formed by vortexing for 5 min) with and without adding *Exo*I.
(TIF)

Figure S3 **Single-exon amplification in emulsions, in separate reactions for each** ***TP53*** **exon, using a 5% dilution of mutation-containing DNA into wild-type DNA.** **A**. conventional PCR in emulsion. **B**. temperature-tolerant fast COLD-PCR in emulsion.
(TIF)

Figure S4 **TT-fast-eCOLD-PCR multiplex (** ***TP53*** **exons 6–9) amplification in solution (without emulsion formation), using a 5% dilution of mutation-containing DNA into wild-type DNA. A**. conventional multiplex PCR. **B**. temperature-tolerant fast COLD-PCR multiplex in solution.
(TIF)
