# Supplementary figures and images for: Enrichment of Mutations in Multiple DNA Sequences Using COLD-PCR in Emulsion
Source: PLoS One. 2012 Dec 6;7(12):e51362. doi: 10.1371/journal.pone.0051362 (PMC3516544; doi:10.1371/journal.pone.0051362)

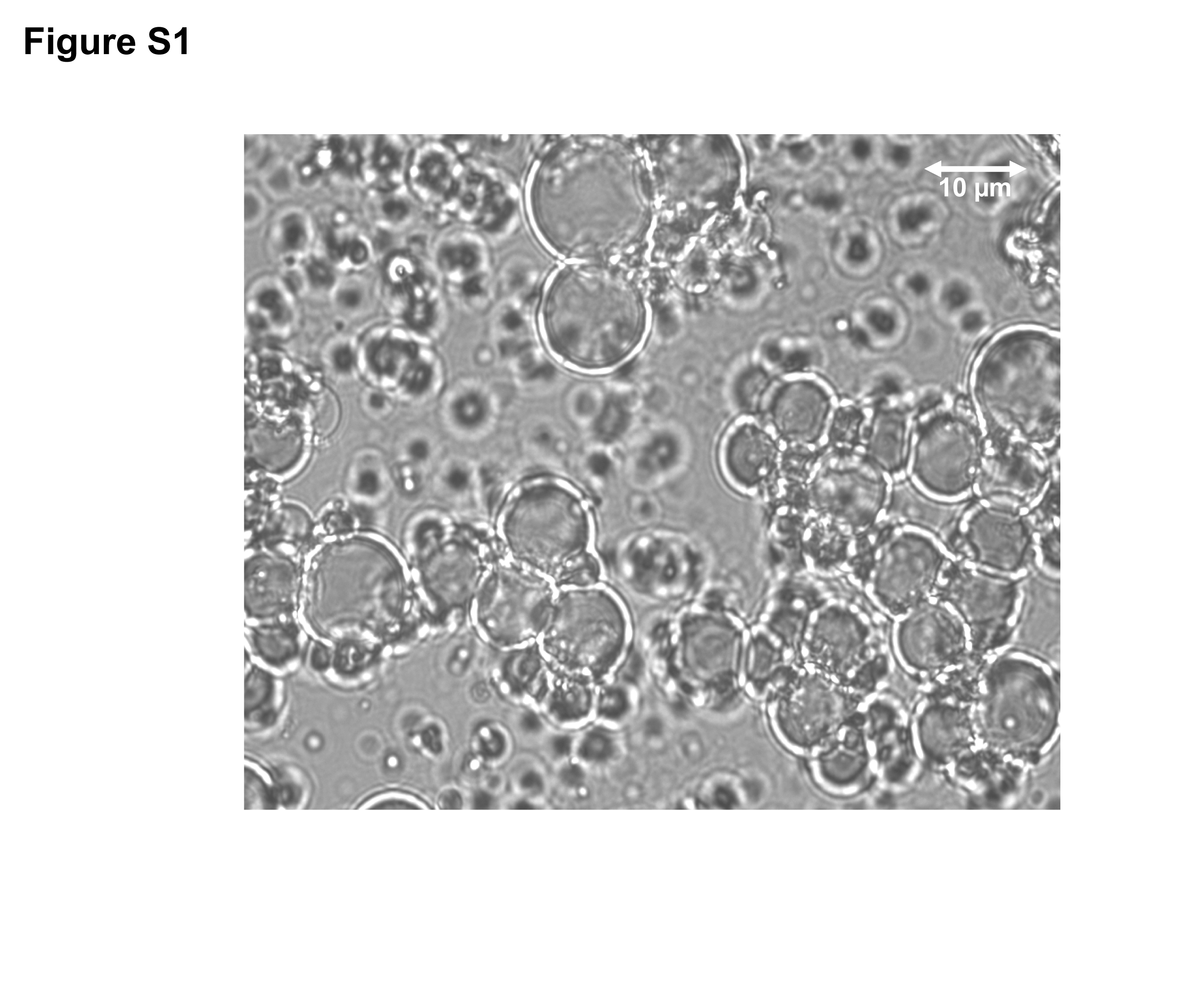

Supplement: Figure S1 — Water in oil emulsion at 100× magnification with a Zeiss Axioimager Z1 fluorescent microscope (Carl Zeiss Microscopy, GmbH, Germany) after five minutes of vortexing. (TIF) [file pone.0051362.s001.tif]

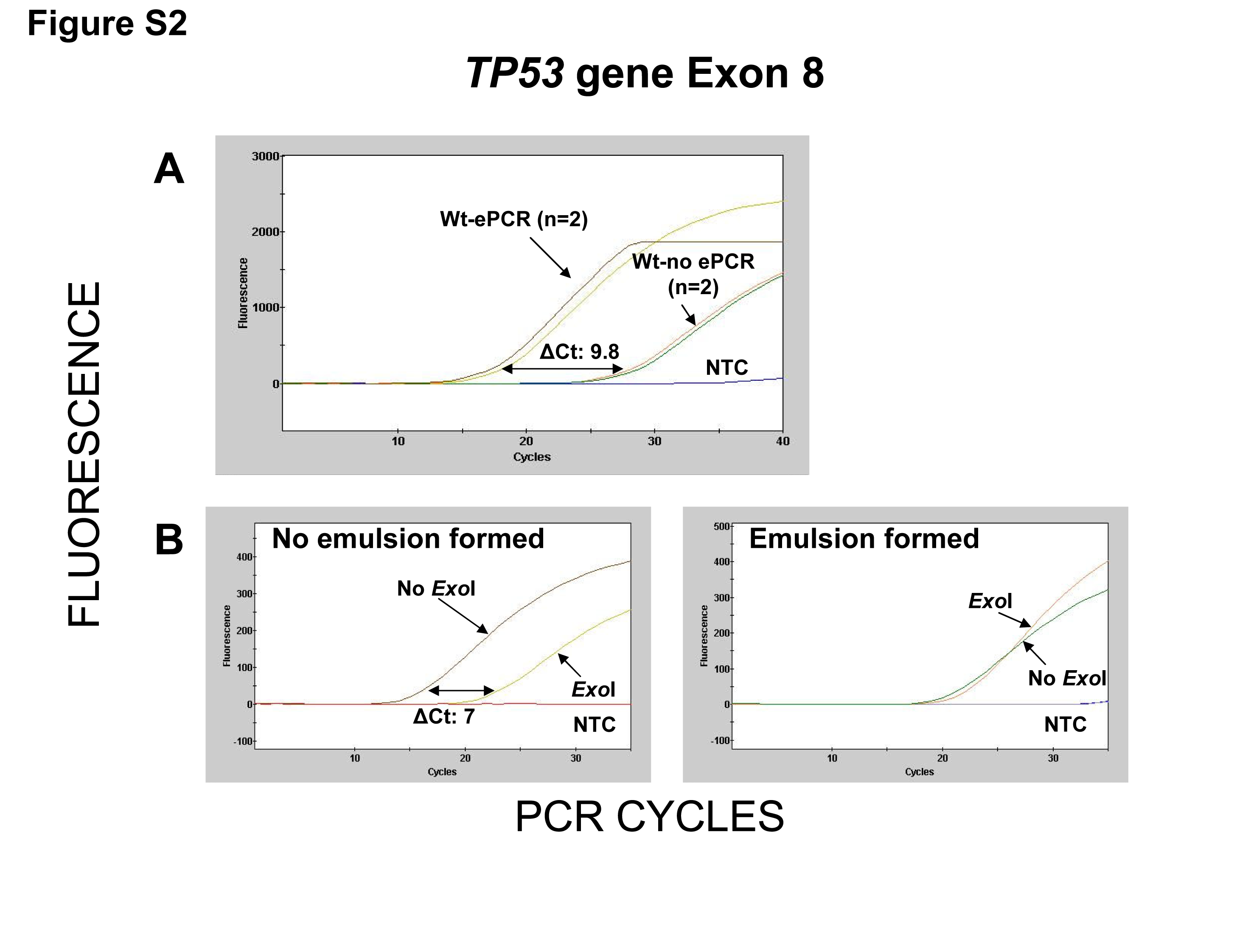

Supplement: Figure S2 — Validation of emulsion-PCR. Panel A: DNA amplified by emulsion PCR (ePCR) or where ePCR amplification was omitted, was evaluated by an additional nested, real-time qPCR using LCGreen (duplicate reactions). Panel B: Effect of adding exonuclease I (ExoI) in the sample prior to ePCR, with and without emulsion formation, as evaluated by nested, qPCR. Left : aqueous and oil phase without emulsion formation (gently mixed) with and without ExoI. Right : emulsion (formed by vortexing for 5 min) with and without adding ExoI. (TIF) [file pone.0051362.s002.tif]

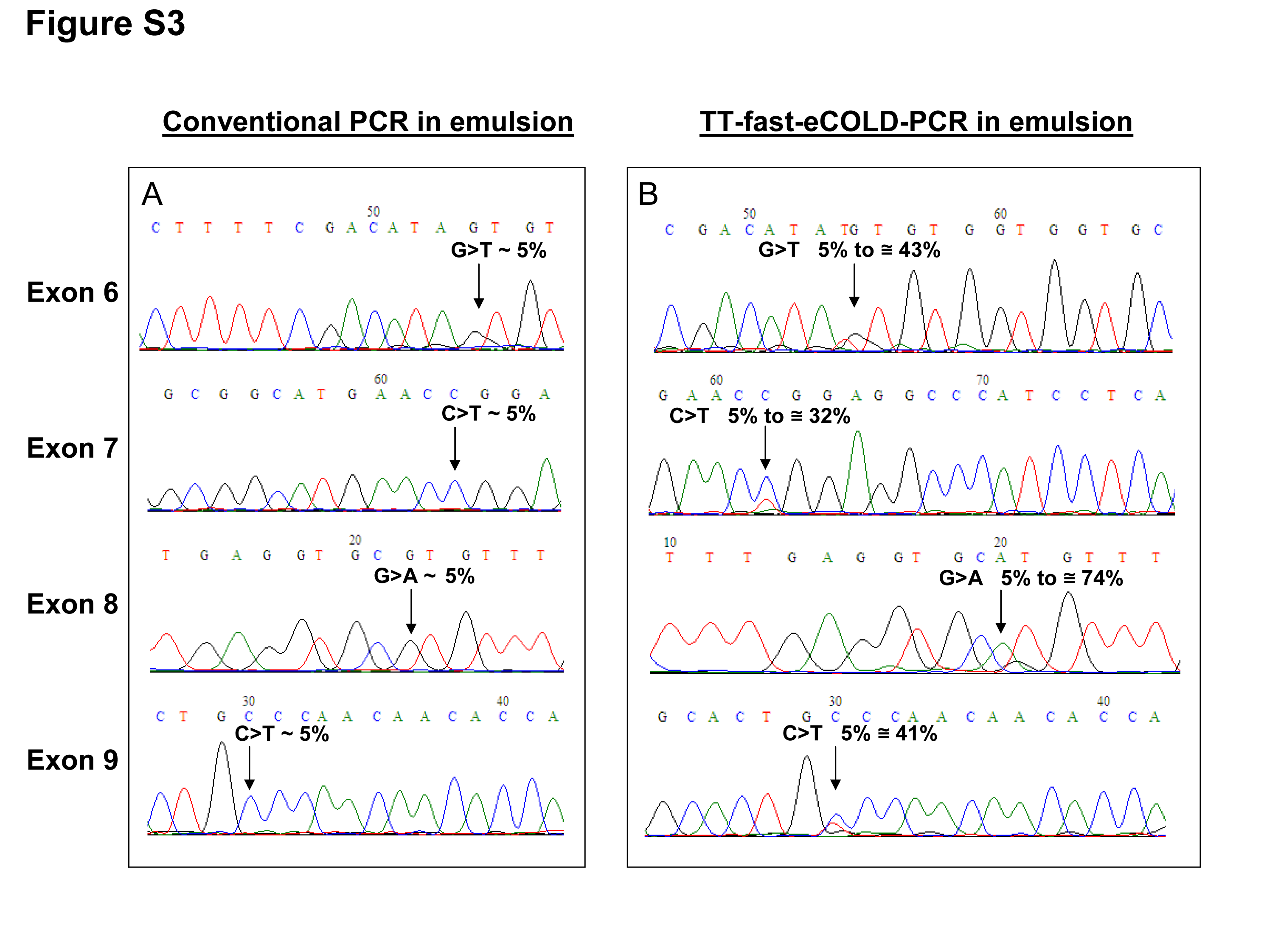

Supplement: Figure S3 — Single-exon amplification in emulsions, in separate reactions for each TP53 exon, using a 5% dilution of mutation-containing DNA into wild-type DNA. A. conventional PCR in emulsion. B. temperature-tolerant fast COLD-PCR in emulsion. (TIF) [file pone.0051362.s003.tif]

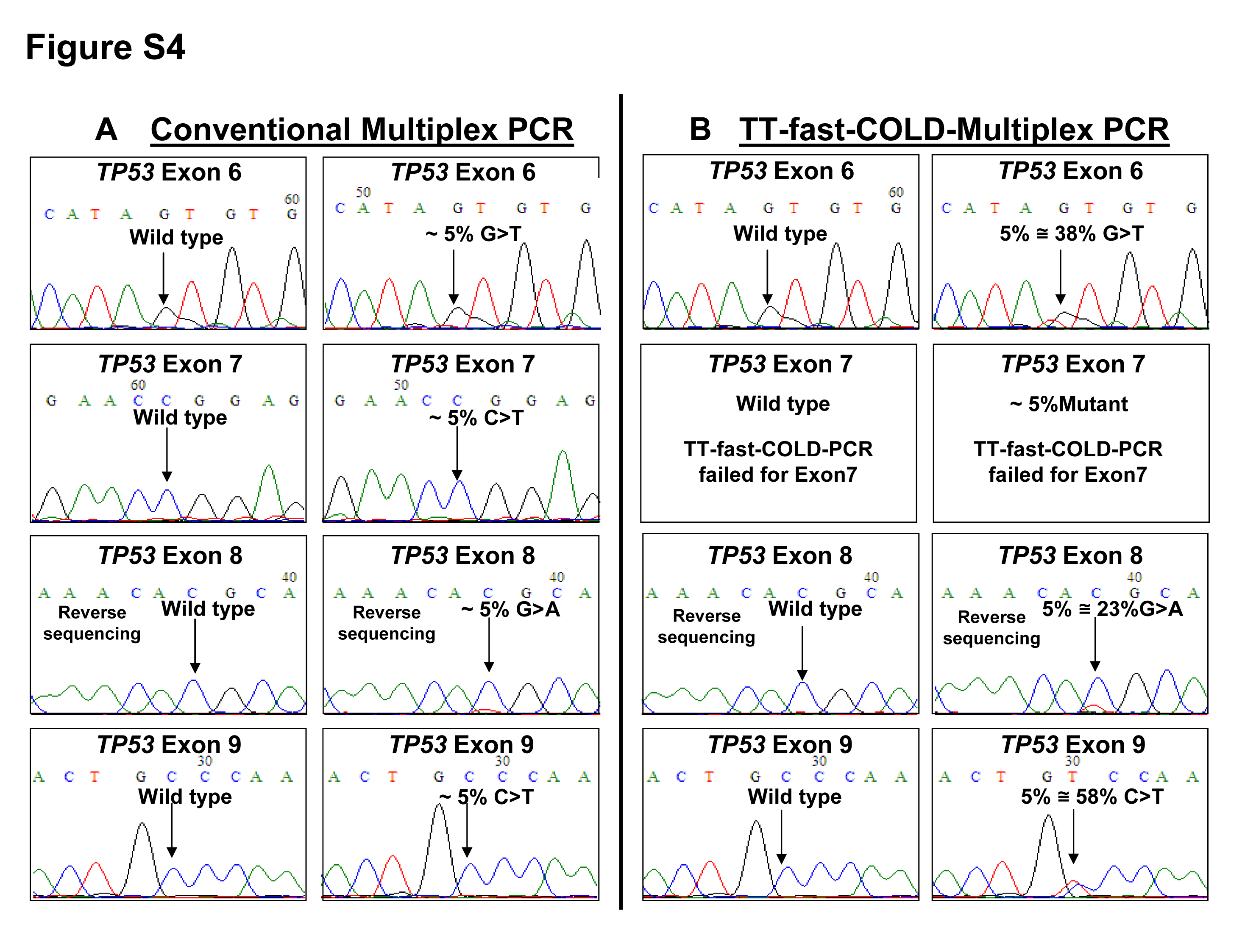

Supplement: Figure S4 — TT-fast-eCOLD-PCR multiplex ( TP53 exons 6–9) amplification in solution (without emulsion formation), using a 5% dilution of mutation-containing DNA into wild-type DNA. A. conventional multiplex PCR. B. temperature-tolerant fast COLD-PCR multiplex in solution. (TIF) [file pone.0051362.s004.tif]
